# Feasibility and first results of a prospective cohort study to investigate cisplatin-associated ototoxicity amongst cancer patients in South Africa

**DOI:** 10.1186/s12885-021-08567-0

**Published:** 2021-07-16

**Authors:** Jessica Paken, Cyril D. Govender, Mershen Pillay, Birhanu T. Ayele, Vikash Sewram

**Affiliations:** 1 Discipline of Audiology, School of Health Sciences, University of KwaZulu-Natal, Private Bag X54001, Durban, 4000 South Africa; 2 African Cancer Institute, Department of Global Health, Division of Epidemiology and Biostatistics, Faculty of Medicine and Health Sciences, Stellenbosch University, P.O. Box 241, Cape Town, 8000 South Africa

**Keywords:** Cisplatin, Ototoxicity, Cervical Cancer, South Africa

## Abstract

**Background:**

Cervical cancer, one of the most common cancers affecting females in South Africa, commonly requires a cisplatin-based-treatment regimen, which has been associated with ototoxic side effects. However, cisplatin-associated ototoxicity is largely under-reported in South Africa, despite its impact of hearing loss having serious overt ramifications on the quality of life of these patients. Hence, a prospective cohort study was undertaken to assess the audiological changes in female cervical cancer patients receiving cisplatin therapy.

**Objective:**

To present details of the feasibility study and initial results on hearing patterns in cervical cancer patients receiving cisplatin chemotherapy. .

**Methods:**

Fifty cervical cancer patients commencing with cisplatin chemotherapy underwent audiological assessments at a hospital in South Africa at various time intervals. Assessments included case history, otoscopic examination, immittance audiometry, pure tone audiometry (including high-frequency audiometry), speech audiometry, and distortion product otoacoustic emission testing. Data analysis involved the use of descriptive statistics and the Cochran-Armitage trend test for a linear trend in proportions.

**Results:**

Fifty participants, aged between 32 and 79 years (Mean: 53 years; SD = 11.00), were recruited. Clinical findings revealed an incidence of 100% ototoxic hearing loss at the one-month post-treatment, i.e., 98%  after three cycles of cisplatin and 2%  at one-month post-chemotherapy. Sensorineural hearing loss and high-frequency tinnitus were most common. Deterioration in hearing thresholds was more evident in the extended high-frequency range, with the number of “no-responses,” from 11,200 Hz to 20,000 Hz, increasing with each successive audiological evaluation. This study further indicated that recruitment and follow-up of study participants within a limited resource setting are possible. However, cognizance must be given to a multidisciplinary approach and constant engagement with participants through regular contact either telephonically or via a short-message-system.

**Conclusion:**

Exposure to cisplatin treatment contributed to hearing loss in females with cervical cancer, highlighting the need for ototoxicity monitoring during chemotherapy treatments. Furthermore, the results indicate that it is possible to conduct prospective cohort studies, using a multidisciplinary approach in limited-resource environments with appropriate planning and training strategies, as this study was able to achieve its aim successfully.

## Background

Cervical cancer is the second most prevalent cancer, accounting for over 20% of all female cancer types in Africa [1]. In South Africa, it is the most common cancer amongst the black female population, with an age-standardized incidence rate of 28.25 per 100,000 and a lifetime risk of 1 in 33 [2]. Treatment of cervical cancer may include surgery, radiotherapy, chemotherapy, or a combination of modalities based on International Federation of Gynecology and Obstetrics (FIGO) stage [3]. Concurrent chemoradiotherapy using weekly cisplatin as the chemotherapeutic agent is the standard of care as an outpatient therapy for locally advanced cervical cancer (LACC), commonly diagnosed in our setting [4].

Cisplatin, however, possesses ototoxic properties [5], where patients exposed to this drug experience loss of hearing and/or vestibular function resulting from the functional and cellular damage of the inner ear [6], for which no medical treatment or prevention currently exists [7]. As cervical cancer is an acquired immunodeficiency syndrome (AIDS)-defining illness, this condition adds further distress for women who are on anti-retroviral therapy (ART), which also has been reported to have ototoxic effects [8]. Affected women generally present with a host of chronic conditions for which they are then prescribed other classes of ototoxic drugs, which can include but not limited to aminoglycosides, loop diuretics, quinine, and non-steroidal anti-inflammatory drugs [9].

The impact of hearing loss on communication has serious overt ramifications on an individual’s quality of life [10]. Consequently, effective communication is often hindered, where the hearing-impaired individual may experience social, emotional, and vocational difficulties [11]. This may be the case for patients with cervical cancer who already present with various symptoms, who now also experience a reduced hearing sensitivity to the point that they miss relevant information regarding treatment regimens. Therefore, it is crucial that the incidence and severity of cisplatin-associated ototoxicity are known, so that the extent of this additional comorbidity may be acknowledged, and appropriate interventions set in place.

The incidence of cisplatin-associated ototoxicity ranges from 13 to 96% [12] and varies due to several factors. For example, differences in treatment dosages, both within a cycle and the total amount administered over multiple cycles (accumulative dose), the time interval between treatment courses, method of administration, treatment duration, as well as differences in the patient population, such as patient demographics, physiology, and clinical factors, all of which accounts for these discrepancies. A review revealed that despite many international studies focusing on cisplatin-associated ototoxicity, only one study (retrospective cross-sectional) reported on its incidence in SA [13]. This study revealed that 55% of the patients developed ototoxicity while receiving high-dose (≥60 mg/m^2^) cisplatin treatment; however, it is unclear to what extent previous noise exposure, pre-existing hearing loss or the use of previous ototoxic medication may have impacted this condition.

The longitudinal nature and degree of variability in hearing loss, after initiation of chemotherapy, required that a “fit-for-purpose” prospective study be conducted. This would allow scrutiny of audiological status prior to, during and at intervals after treatment, in the presence of confounders to exposures but also among other variables of interest. This approach also sought to reduce measurement error and avoid bias resulting from missing data associated with retrospective chart audits.

The utilization of this type of study design is relatively uncommon in the audiology domain in SA, hence we embarked on a planning and feasibility phase. Here, we present the details of the feasibility study and initial results on the hearing patterns of cervical cancer patients during the course of cisplatin chemotherapy.

## Methods

### Aim

To present details of the feasibility study and initial results on hearing patterns in cervical cancer patients receiving cisplatin chemotherapy.

### Study design

A prospective cohort study design was reported on.

### Setting

The study was conducted at a referral hospital offering tertiary services in KwaZulu-Natal (KZN), South Africa (SA), as defined in the regulations relating to categories of hospitals in SA. This hospital is also one of the main referral centers for cancer patients and houses an audiology department.

### Population

The study population comprised of female patients diagnosed with cervical cancer receiving a cisplatin-based chemotherapeutic treatment regimen.

### Sampling

Females who were 18 years or older, with an incident diagnosis of cervical cancer, commencing with the first cycle of chemotherapy were invited to participate in the study. Patients presenting with profound hearing loss at baseline assessment, or those who had previously received cisplatin chemotherapy, or had a history of medical conditions such as tuberculosis and malaria were excluded, as the treatment for such conditions often require the use of ototoxic drugs. However, since cervical cancer is considered an AIDs-defining illness, all participants were tested for their HIV status, and HIV positive women who were on ART were documented.

### Data collection procedures

Data was collected over a 10-month period during which 50 participants underwent audiological evaluations prior to chemotherapy initiation, at the beginning of the fourth cycle and at one-month post-treatment.

Staff at the oncology department (doctors, nurses, and radiotherapists) willingly assisted with the recruitment of participants. In addition, the primary researcher checked the statistics report of the oncology department weekly to ensure that no patients were missed. Individual data collection commenced following informed consent.

A detailed case history interview was conducted at each assessment to obtain information on participant’s audiological and otological status, medical conditions, medication, noise exposure, and communicative abilities related to audition. Information on the risk factors and symptoms of ototoxic hearing loss was also documented during the case history interview, in addition to patient contact details (mobile phone numbers of participants and close relatives) for the purpose of follow-up. Participants were reminded of their appointments, either telephonically or via a short message system (SMS). The monitoring of audiometry was conducted before the fourth cycle of chemotherapy, as this is generally the mid-point of the treatment regimen for patients with cervical cancer.

Appropriate instructions were provided to the participants before the commencement of each audiological procedure. The audiological test, suggested for ototoxicity monitoring by the American Speech And Hearing Association (ASHA, 1994) [14], was utilized and included the following: review of medical file, case history interview, otoscopic examination, immittance audiometry (tympanometry and acoustic reflex threshold testing), pure tone audiometry (air conduction up to 20 kHz, and bone conduction), speech reception threshold testing, speech discrimination testing and distortion product otoacoustic emission testing [14, 15]. A qualified technician calibrated all equipment, while the researcher conducted daily biological calibrations before data collection.

To accommodate local prerequisites, all correspondence with participants was translated into isiZulu, which is the local language spoken by the majority of the population in KwaZulu-Natal. Therefore, information and informed consent documents, case history questionnaires, and instructions for audiological procedures were available in both English and isiZulu. An isiZulu linguist verified translations. The primary investigator was also able to converse in isiZulu, having completed a course at university. The entire audiological test took a maximum of 45 min to complete.

All audiometric test results were interpreted according to the norms prescribed in the literature [16]. On identification of a significant ototoxic hearing loss, an audiological retest was conducted within 24 h to verify the change [14]. The presence of a significant ototoxic change was determined using the ASHA Association [14] criteria, defined as follows:

(a) ≥ 20 dB decrease at any one test frequency,

(b) ≥10 dB decrease at any two adjacent frequencies, or.

(c) loss of response at three consecutive frequencies where responses were previously obtained.

On identification of a reduction in hearing ability, participants were counseled regarding treatment options such as compensatory communication strategies, as well as rehabilitation technology options, and referred to the necessary medical personnel, i.e., either the oncologist, ear-nose-throat specialist, or the psychologist. Participants were also encouraged to refrain from noisy environments, as this would exacerbate the experienced hearing loss.

### Data analysis

Feasibility issues were evaluated by considering the eligibility and accrual of participants, the capacity of the study site; follow up rates, and the results obtained in the audiological evaluation of the participants. Descriptive statistics were used to summarize demographic and clinical characteristics of the study participants, where mean and standard deviation (SD) was used for normally distributed variables and median and interquartile range (IQR) for non-normal variables. Shapiro Wilk test was used to assess normality. Categorical variables were presented using frequency and percentages and comparisons were performed using the Fisher’s exact test. The Cochran-Armitage trend test for trend in proportions was used to examine changes in case history findings over time. Ototoxic hearing loss was determined using the ASHA (1994) [14] guidelines. Statistical significance was accepted at *p* < 0.05 for all statistical tests. All data were analyzed using the STATA 15 software (StataCorp. 2017, Texas, USA).

## Results

### Participant characteristics

Approximately five of the 30 patients diagnosed with cervical cancer at the study site each month, commenced with chemotherapy. As four participants did not meet the selection criteria, the study sample comprised 50 females. As indicated in Table 1, the study population comprised of three ethnic groups (as categorized by the South African National Cancer Registry) between 32 and 79 years of age, with the mean age being 53 years (SD = 11).

**Table 1:** Demographic and clinical characteristics of cervical cancer patients (*n* = 50)

Characteristic	n (%)
*Age (years)*	
≤ 40	8 (16)
41–50	8 (16)
51–60	21 (42)
> 60	13 (26)
*Ethnicity*	
Black African	44 (88)
Indian/Asian	3 (6)
Coloured	3 (6)
*Stage of cancer*	
I A	1 (2)
II A	7 (14)
II B	25 (50)
III A	4 (8)
III B	13 (26)
*Comorbidities*	
None	11 (22)
HIV infection only	20 (40)
Hypertension only	10 (20)
Diabetes only	2 (4)
Diabetes and hypertension	4 (8)
HIV and hypertension	2 (4)
HIV and Epilepsy	1 (2)
*Cisplatin Dosage*	
Mid-cycle (150 mg/m^2^)	50 (100)
One-month post-treatment follow up	
150 mg/m^2^	8 (16)
200 mg/m^2^	14 (28)
250 mg/m^2^	10 (20)
300 mg/m^2^	18 (36)

The majority of participants in this cohort were black African women (*n* = 44) (88%), with the remaining six participants (12%) comprising of an equal distribution of the Indian/Asian and Coloured women, respectively. Fifty percent (*n* = 25) of the participants were diagnosed with Stage IIB cervical cancer, with stage IA2 being the least common, as diagnosed in one patient (2%). Twenty-three (46%) females were HIV positive. All participants received concomitant chemoradiation therapy. Chemotherapy consisted of cisplatin (50 mg/m^2^ body surface weekly for six cycles) in combination with dexamethasone (48 mg) and ondansetron (24 mg). All participants received 3 cycles of cisplatin chemotherapy (cumulative dosage of 150 mg/m^2^) at mid- cycle. Eight patients (16%) were found to have discontinued treatment after the third cycle, having received a cumulative cisplatin dosage of 150 mg/m^2^, while the remaining participants completed their cycles to varying degrees as indicated in Table 1. All participants presented with normal renal function during the course of treatment.

### Increasing complaints of reduced hearing sensitivity and tinnitus

A summary of the case history enquiry at each audiological evaluation is presented in Table 2.

**Table 2:** Summary of case history findings (*N* = 50)

Patient Complaints	Baseline	Mid-cycle	1-month post-treatment	*p*-value
*Repeated Ear infections*	*2 (4)*	*2 (4)*	*2 (4)*	*0.988*
Bilateral	0	0	0	
Left ear only	1 (50)	1 (50)	1 (50)	
Right	1 (50)	1 (50)	1 (50)	
*Reduced hearing*	*7 (14)*	*8 (16)*	*11 (22)*	*0.36*
Bilateral	4 (57.1)	5 (62.5)	5 (45.4)	
Left ear only	1 (14.3)	1 (12.5)	3 (27.3)	
Right ear only	2 (28.6)	2 (25)	3 (27.3)	
*Otalgia*	*3 (6)*	*8 (16)*	*5 (10)*	*0.52*
*Tinnitus*	*21 (42)*	*26 (52)*	*17 (34)*	*0.42*
Bilateral	10 (47.6)	14 (53.9)	11 (64.7)	
Left ear only	8 (38.1)	7 (26.9)	1 (5.9)	
Right ear only	1 (4.8)	3 (11.5)	4 (23.5)	
Head	2 (9.5)	2 (7.7)	1 (5.9)	

*Cochran-Armitage test for a linear trend in proportions

**Fig. 1:**
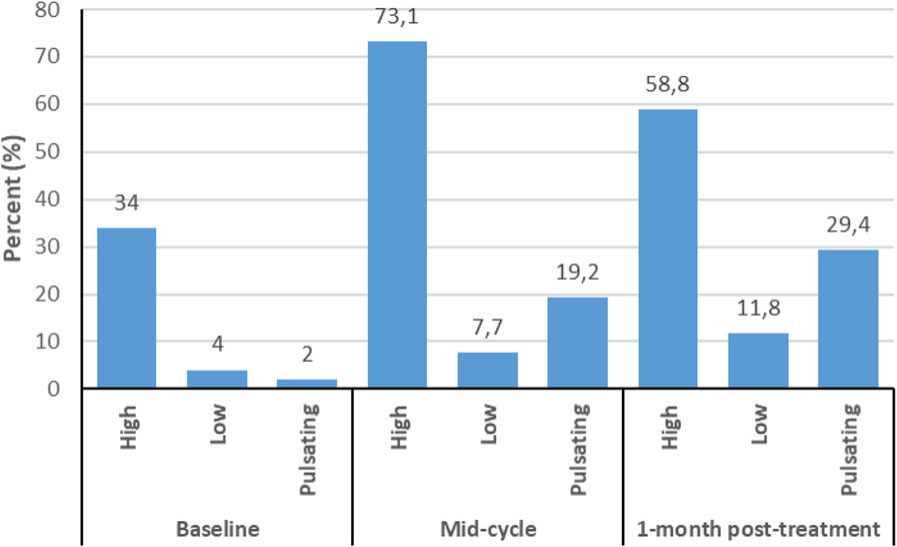
Percent distribution and description of participants with tinnitus (*n* = 50)

At baseline, seven (14%) participants reported reduced hearing sensitivity, with the number of complaints increasing at each follow-up, although the increase was not significant (*p* = 0.36) (Table 2). Bilateral hearing difficulties were more frequently reported than unilateral. Tinnitus was found to be the most common otologic symptom experienced by participants, i.e., 21 (42%) at baseline, 26 (52%) after three cycles of chemotherapy, and 17 (34%) at the one-month follow-up (Table 2). Reports of tinnitus increased after the three cycles of chemotherapy and decreased at the one-month post-chemotherapy audiological evaluation. Results show no evidence of significant changes over time for reported tinnitus (*p* = 0.42) (Table 2). A similar pattern is seen with the reports of otalgia. High-frequency tinnitus was the most commonly reported at all audiological evaluations, as reflected in Fig. 1. Only two participants reported previous repeated ear infections on all successive evaluation times, i.e., one on the right ear and the other on the left ear.

### Deterioration in hearing thresholds more evident in the extended high-frequency range

Otoscopic examination findings were normal in 48 (96%) participants bilaterally, with a tympanic membrane perforation visualized in the left ear of one participant and the right ear of another. Tympanometry was consistent with Type A tympanograms being obtained in the right ear of 49 participants (98%) and the left ear of 49 participants (98%).

Clinical findings, as reflected in Table 3, showed an increase, although not statistically significant (p= > 0.1), in the number of participants presenting with hearing loss from baseline to mid-cycle chemotherapy.

**Table 3:** Summary of clinical findings (*n* = 50)

Clinical Findings	Baseline	Mid-cycle	One month post-treatment	*p*-value (Test for trend)*
*Hearing loss*^*a*^				
Bilateral	11 (22)	16 (32)	18 (36)	0.13
Left ear only	4 (8)	3 (6)	1 (2)	0.17
Right ear only	1 (2)	1 (2)	2 (4)	0.56
*Type of hearing loss*				
Sensorineural	15 (30)	19 (38)	19 (38)	–
Conductive	0	0	0	–
Mixed	1 (2)	1 (2)	1 (2)	–
*Ototoxic hearing loss*^*b*^	*0*	*49 (98) *	*1 (2) *	
Bilateral				
Left ear only				
Right ear only				

*Cochran-Armitage test for a linear trend in proportions

^a^The presence of a hearing loss was determined using the Silman and Silverman’s (1991) magnitude of hearing impairment. If thresholds exceeded 25 dB at more than three frequencies in the conventional frequency range, it was classified as a hearing loss

^b^Ototoxic hearing loss was determined using the ASHA (1994) guidelines, which indicates the following: (a) ≥ 20 dB decrease at any one test frequency, (b) ≥10 dB decrease at any two adjacent frequencies, or (c) loss of response at three consecutive frequencies where responses were previously obtained

**Fig. 2:**
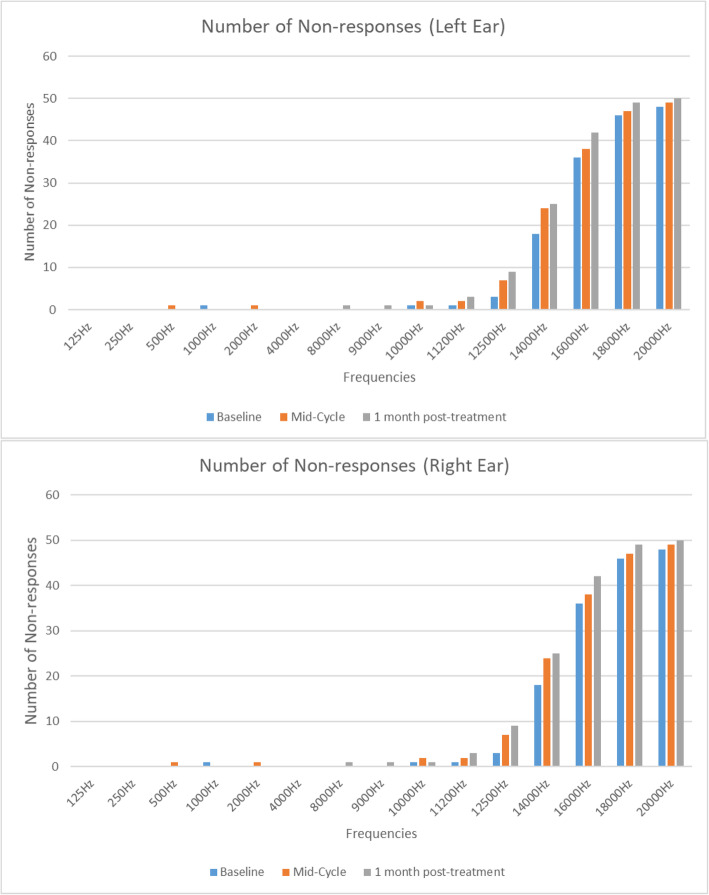
Number of “NO RESPONSES” at the different frequencies (*n* = 50)

A steady increase in the number of participants with hearing loss, while not significant, is evident at each successive audiological evaluation. Sensorineural hearing loss was the most common type of hearing loss diagnosed at each audiological evaluation, with only one participant (2%) presenting with a mixed hearing loss (Table 3). Ototoxic hearing loss was evident in 49 (98%) participants after three cycles of chemotherapy and 1 ( 2%) participant at the one-month post chemotherapy evaluation (Table 3). An increase in the number of ‘no-responses’ at each subsequent audiological evaluation was evident from 11,200 Hz to 20,000 Hz, as reflected in Fig. 2; thus, revealing the extended high-frequency range to be most affected.

**Fig. 3:**
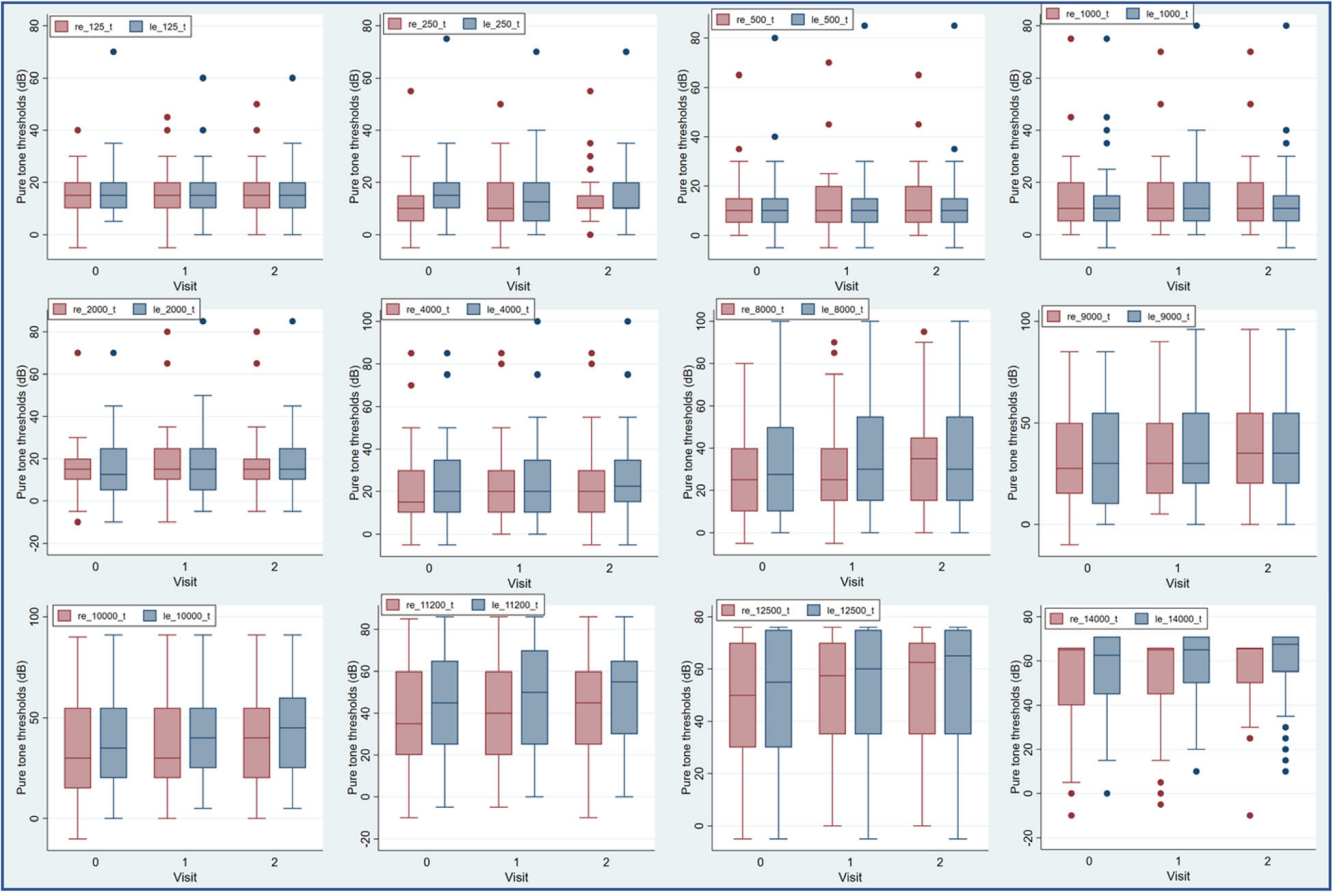
Distribution of pure-tone hearing thresholds in right and left ears prior to chemotherapy initiation (visit 0), mid-cycle (visit 1) and post-treatment (visit 2) measured at frequencies ranging from 125 Hz to 14,000 Hz. The bottom and top of the box are the 25th and 75th centiles and the line in the box is the median; the lower and upper lines represent the minimum and maximum values. The distribution, depicted by the median, displays an increasing trend in hearing threshold bilaterally between 8000 Hz – 14,000 Hz.Footnote: The medians of 16,000, 18,000 and 20,000 Hz are not presented on the table due to the large number of ‘No Responses’ (responses beyond the limits of the audiometer) at these frequencies

There is a progression of hearing loss over time, as reflected by the increase in median pure tone thresholds from the baseline assessment to each of the follow-up evaluations, especially evident from 8000 Hz to 14,000 Hz bilaterally (Fig. 3).

At one month post treatment follow-up, 11/23 (47.8%) HIV positive participants progressed to Grade 1 (*n* = 8), Grade 2(*n* = 3) stages of hearing loss respectively, based on the NCI-CTCAE Grading Scale [17]. In comparison, 8/27 (29.6%) HIV-negative participants progressed to Grade 1 (*n* = 7), Grade 2 (*n* = 1) stages of hearing loss respectively. The Fisher’s exact test revealed no significant difference (*p* = 0.25), possibly attributable to the small sample size and limited follow-up.

**Fig. 4:**
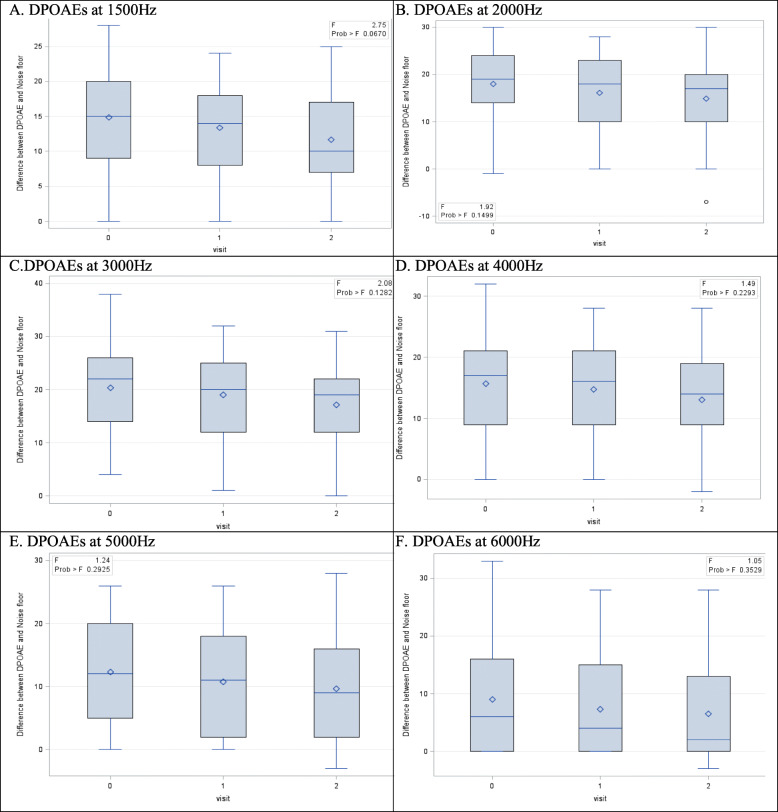
Box plots of OAEs for the Right Ear (Repeated ANOVA with p-value) (*n* = 49). Data are presented as median and IQR

**Fig. 5:**
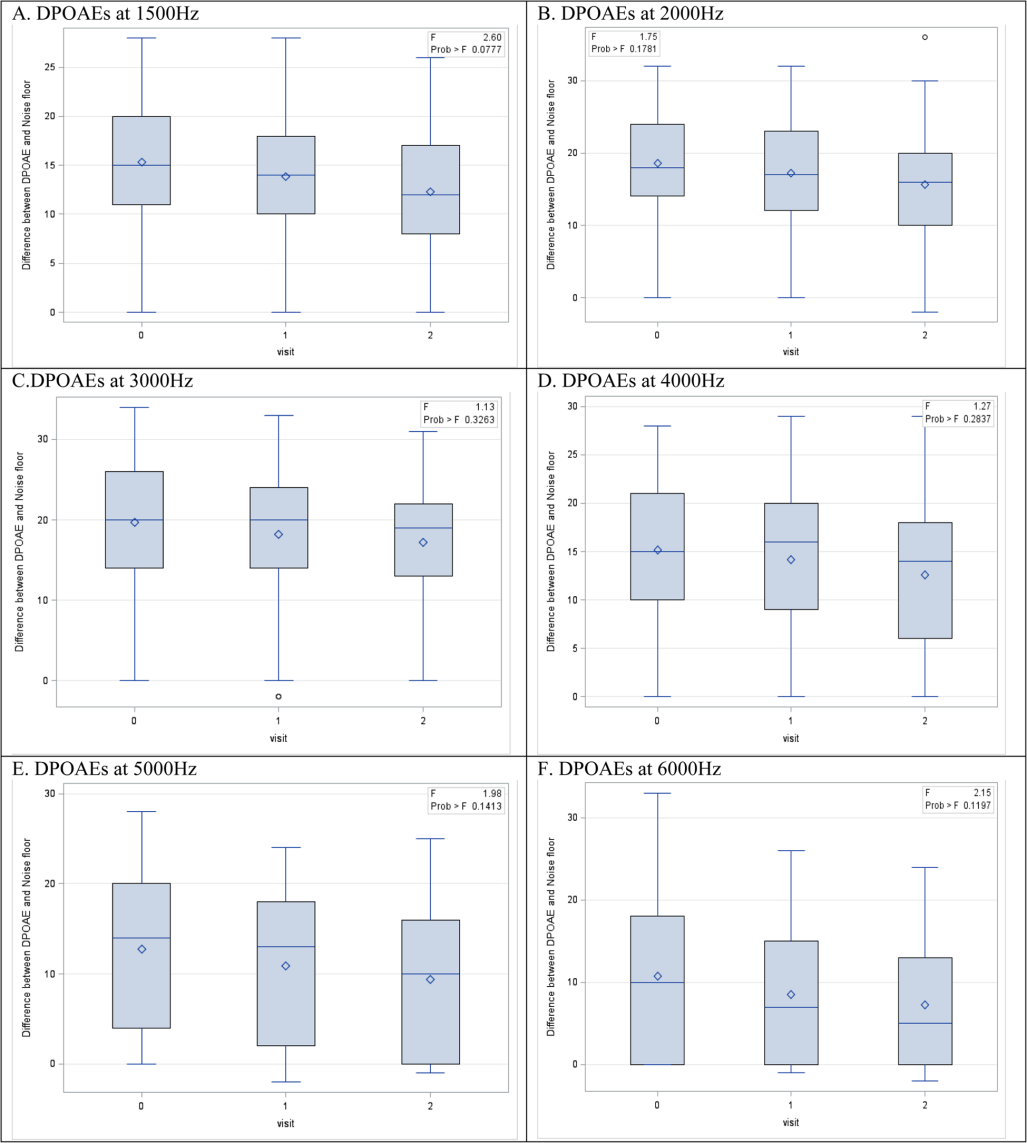
Box plots of OAEs for the Left Ear (Repeated ANOVA with *p*-value) (*n* = 49). Data are presented as median and IQR

Figure 4 and Fig. 5 revealed a decrease in the difference between the DPOAE and the noise floor for all frequencies tested in the right ear of 49 participants and the left ear of 49 participants, respectively. However, this difference was not found to be statistically significant (*p* > 0.05).

## Discussion

Our pilot data has demonstrated the feasibility of undertaking such a study in a resource-constrained environment, provided that the basic principles of epidemiology is adhered to and that the project is underpinned by strong intersectoral collaboration and multidisciplinary team effort. It was possible to accrue and follow up patients through study sensitization and regular contact, respectively. We observed that employing a multidisciplinary approach to the recruitment of participants was beneficial and therefore required ‘buy-in’ from the relevant healthcare personnel. This would only be possible if the healthcare personnel has been informed of the purpose of the study and the benefit to the patient population. The study site, being the main referral center for oncology services, was deemed suitable due to the high incidence of cervical cancer within a large catchment area, allowing for the generalizability of data. The hospital also has an oncology department, as well as an audiology department for robust and scientific assessment, hence site capacity is an essential evaluation parameter for such studies, given the larger scale investments that are placed into the conduct of prospective cohort studies. An audiology department away from the hospital can result in higher attrition, as patients may be too ill or may not have the funds to travel for follow up visits to additional sites. Therefore, an assessment of the environment is essential to ensure proper planning concerning patient recruitment and their time and travel management.

The selection of patients with locally advanced cervical cancer was appropriate to study the impact of cisplatin on hearing loss, in this setting, given the high incidence of cervical cancer and the synchronicity of the treatment protocol. These assessment parameters allow for a higher patient population and greater probability of the inclusion criteria being met within the study period.

Patient follow-up messages and appointment reminders were communicated through mobile phones via voice contact short message system (SMS). This has highlighted the growing influence of mobile phones as a tool for research and public health intervention and adds to the growing body of evidence on the value of mobile devices within health platforms [18, 19, 20].

Our study also showed an increasing number of women with complaints of reduced hearing sensitivity and clinical hearing loss at each successive audiological evaluation.  All participants in the study presented with significant ototoxic change by the one-month post-treatment evaluation, similar to studies conducted in India [5, 21]. The incidence of  ototoxicity in the current study is higher as compared to other studies [22, 23, 24, 25]. These differences can be ascribed to methodology differences as the current study utilized high-frequency audiometry during the audiological evaluations, whereas Nitz et al. (2013) [24], and Nagy et al. (1999) [25], utilized pure tone audiometry in the conventional frequency range. Furthermore, diseases such as diabetes [26], hypertension [27] and HIV as well as treatment for HIV [28] may also negatively influence hearing, as seen in the current study with 15 participants presenting with hearing loss at baseline. Complaints of reduced hearing sensitivity were much lower than that revealed by the baseline audiological assessment, indicating that the participants may have gradually adjusted to the reduced hearing sensitivity due to the loss being gradual. This is generally seen in presbycusis and is consistent with the age characteristics of our study population.

As is seen in ototoxic hearing loss, the findings of our feasibility study revealed sensorineural hearing loss to be most common, due to the structures of the inner ear being most susceptible to damage by cisplatin chemotherapy; with apoptotic degeneration of the hair cell in the organ of Corti being most prominent [29]. In addition, the extended high-frequency range appeared to be more affected. This is consistent with literature stating that the outer hair cells in the basal turn of the cochlea are most affected, resulting in an initial elevation of high-frequency audiometric thresholds, followed by a progressive loss in the lower frequencies with continued therapy [6, 30]. This, therefore, highlights the need for the inclusion of high-frequency audiometry in the ototoxicity-monitoring programme.

The most common otologic symptom experienced by the study participants was tinnitus. With no national averages available in the country and 42% of participants reporting tinnitus at baseline, the current authors postulate that an increase in the stress and anxiety associated with receiving cisplatin chemotherapy, as well as thoughts about their prognosis, may have resulted in tinnitus. Hasson et al. [31] reported a linear association between tinnitus and the magnitude and duration of stress, which is consistent with the pattern observed in the current study, whereby the number of participants reporting tinnitus increased during treatment but decreased post-treatment. The resolution of tinnitus is in keeping with Bokemeyer et al. [32], who reported that tinnitus resolved or decreased in some patients receiving chemotherapy after a median duration of 6 months (range 1–18 months). Melamed et al.  [33] and Reddel et al. [34] also indicated that while the reversibility of tinnitus was common, threshold abnormalities persisted.

Furthermore, 23 (46%) of the participants were HIV positive and receiving ART thus, indicating that it is not feasible to exclude patients with HIV in this cervical cancer population, as it may substantially increase the duration of recruitment. In light of this, it is essential to include information on HIV status, additional comorbidities and other potential ototoxic drugs as confounders when assessing cisplatin-associated ototoxicity. Studies indicate that some diseases such as hypoalbuminemia, anemia [35], renal insufficiency [32], and diabetes [36] are considered to place patients at a higher risk for ototoxicity. Additional factors that may increase the risk for ototoxicity include cumulative dosage, the number of cycles administered, method of administration, exposure to high levels of concomitant noise, chemicals and other ototoxic medication [36], genetic risk factors (megalin and glutathione S-transferases gene ﻿﻿polymorphism﻿) [37], pre-exposure hearing ability and age [38, 39].

## Conclusion

Our study has demonstrated that a prospective cohort study within the audiological domain is feasible in our setting, allowing for the collection of relevant medical and audiological information to investigate the hearing patterns of cervical cancer patients. This experience communicated the need for this study and may bear relevance to other resource-limited settings with a high cervical cancer burden where audiologists may wish to study hearing loss in similar cohorts, so long as the relevant expertise and support is available.

## Data Availability

The data that supports the findings of this study are available on request from the corresponding author.
